# Development and psychometric testing of an instrument to evaluate cognitive skills of evidence based practice in student health professionals

**DOI:** 10.1186/1472-6920-11-77

**Published:** 2011-10-03

**Authors:** Lucy K Lewis, Marie T Williams, Timothy S Olds

**Affiliations:** 1 School of Health Sciences, University of South Australia, GPO Box 2471, Adelaide, SA, 5001, Australia; 2 Health and Use of Time (HUT) Group, Sansom Institute for Health Research, University of South Australia, GPO Box 2471, Adelaide, SA, 5001, Australia

## Abstract

**Background:**

Health educators need rigorously developed instruments to evaluate cognitive skills relating to evidence based practice (EBP). Previous EBP evaluation instruments have focused on the acquisition and appraisal of the evidence and are largely based in the medical profession. The aim of this study was to develop and validate an EBP evaluation instrument to assess EBP cognitive skills for entry-level health professional disciplines.

**Methods:**

The Fresno test of competence in evidence based medicine was considered in the development of the 'Knowledge of Research Evidence Competencies' instrument (K-REC). The K-REC was reviewed for content validity. Two cohorts of entry-level students were recruited for the pilot study, those who had been exposed to EBP training (physiotherapy students, n = 24), and who had not been exposed to EBP training (human movement students, n = 76). The K-REC was administered to one cohort of students (n = 24) on two testing occasions to evaluate test-retest reliability. Two raters independently scored the first test occasion (n = 24) to evaluate the inter-rater reliability of the marking guidelines. Construct validity was assessed by comparison of the two groups, 'exposed' and 'non-exposed', and the percentage of students achieving a 'pass' score in each of these groups. Item difficulty was established.

**Results:**

Among the 100 participants (24 EBP 'exposed', and 76 EBP 'non-exposed' students), there was a statistically significant (*p *< 0.0001) difference in the total K-REC scores. The test-retest and inter-rater reliability of the individual items and total scores ranged from moderate to excellent (measured by Cohen's Kappa and ICC, range: 0.62 to perfect agreement).

**Conclusions:**

The K-REC instrument is a valid and reliable evaluation instrument of cognitive skills of EBP in entry-level student health professionals. The instrument is quick to disseminate and easy to score, making it a suitable instrument for health educators to employ to evaluate students' knowledge of EBP or in the evaluation of entry-level EBP training.

## Background

The most widely accepted definition of evidence based practice (EBP) involves three components: the integration of the best research evidence with patient values and clinical expertise [1]. Evidence based practice is now well established in the health and social care professions [2]. To date, the main focus of EBP has been on the behaviours (application and practice) of health professionals in the clinical and research environments [3]. More recently, the EBP movement has expanded to include the educational processes (content, delivery and assessment) involved in entry-level training of health professionals [4].

It has been proposed that EBP consists of a five step process [1,5,6]. Previous authors have suggested that this five step model should form the basis not only for health professionals' clinical decision making and practice but for curricular content underpinning EBP training of health professionals [7]. The first four steps of this model involve asking a clinical question, acquiring and appraising the evidence, and applying the evidence into clinical practice. The fifth step encourages individuals to reflect upon the process undertaken in the first four steps. This final step may also provide an opportunity for EBP training providers to undertake formal evaluation procedures to assess the effectiveness of EBP training. The recommendations provided in the Sicily Statement highlight a need for future educational research evaluating EBP training, and the development and application of evaluation instruments to assess EBP training for each of the five steps [7].

A previous systematic review [8] identified 104 instruments for evaluating the effectiveness of EBP training, the majority of which were developed or tested with medical students or practitioners and the minority (n = 13) developed or tested on other health professions. Seven of the 104 instruments identified in this review were recognised as 'Level 1 instruments' (supported by established inter-rater reliability, objective outcome measures, and three or more types of established validity). The 'Fresno test of competence in evidence based medicine' [9] and the Berlin Questionnaire [10] were the only 'Level 1' instruments identified as evaluating all aspects of EBP competence [8]. The 'Fresno test' consists of two clinical scenarios and open ended questions to measure medical professionals' skills and knowledge across the four main steps of the EBP process. The Berlin Questionnaire consists of 15 multiple choice questions which mainly focus on participants' epidemiological skills and knowledge. While the Berlin questionnaire is described as an instrument that assesses EBP competence [8], it only comprehensively evaluates the third step (appraisal) of the EBP process [11].

A further 10 instruments evaluating cognitive EBP skills have been published since the 2006 [8] systematic review (Table 1) [12-21]. Four of these instruments were designed for disciplines external to medicine including complementary medicine [13], nursing (diabetes educators) [15], occupational therapy [20] and physical therapy [21].

**Table 1:** EBP evaluation instruments identified in an updated systematic search (post 2006) and comparison to the Fresno test

Study	Instrument type/name	No. of knowledge items	Psychometric properties	EBP outcomes	EBP content assessed						EBP steps
		**(question type)**			**Research question**	**Search strategy**	**Research design**	**Critical appraisal**	**Statistics**	**Levels of evidence**	

**Ramos et al 2003**[9]	**Fresno test**	**12 (short answer/essay)**	**Inter-rater reliability, IC, content, discriminative and responsive validity**	**K_A_**	**x**	**x**	**x**	**x**	**x**		**1,2,3,4**

Caspi et al 2006 [12]	online survey	10 (m/c)	IC, content validity	K_A_, K_SR_, A					x		3

Krueger 2006 [13]	exam	not reported (m/c)	Content and discriminative validity	K_A_			x	x	x	x	3

Novack et al 2006 [14]	survey	not reported (m/c)	Content and discriminative validity	K_A_			x		x		3

Meyer et al 2007 [15]	not reported	13 (not reported)	Inter-rater reliability, content and responsive validity	K_A_			x		x		3

Shuval et al 2007 [16]	written assignment + online exam	not reported (short answer/essay)	Inter-rater reliability, content validity	K_A_, A, U, B	x	x					1,2

Siriwardena et al 2007 [17]	Manchester short EBM survey	30 (m/c)	IC, content and discriminative validity	K_A_, A		x		x			2, 3

Davis et al 2008 [18]	survey	5 (m/c, short answer)	IC, content, discriminative and responsive validity	K_A_, A	not reported						

Ahmadi-Abhari et al 2008 [19]	survey	6 (m/c)	IC, content and discriminative validity	K_A_, A	not reported						3

McCluskey & Bishop 2009 [20]	Adapted Fresno test of competence in EBP	7 (short answer/essay)	Inter-rater reliability, IC and responsive validity	K_A_	x	x	x	x	x		1,2,3

Tilson 2010 [21]	Modified Fresno Test	13 (short answer/essay)	Inter- and intra-rater reliability, IC, content and discriminative validity	K_A_	x	x	x	x	x		1,2,3, ?4

Bold type indicates the original Fresno test [9], 'x' research evidence competency assessed, ? questionable inclusion.

A Attitudes, B Barriers, IC Internal consistency, K_A _actual knowledge, K_SR _self-reported knowledge, m/c multiple choice, P Perceptions, T/F true/false, U Use.

At the time of the current study (2008), the instrument developed in this study, the 'Knowledge of Research Evidence Competencies' instrument (K-REC) was the first known tool prospectively designed to collect data on cognitive EBP skills of entry-level allied health students. Since the initial literature search and development of the K-REC, two further instruments have been published that evaluated cognitive skills of EBP in the allied health disciplines [20,21]. The 'Adapted Fresno test of competence in evidence-based practice' (AFT) was developed for occupational therapists, based on the first seven items of the Fresno test. Inter-rater reliability (ICC 0.91-0.96) and internal consistency (Cronbach's α 0.74) was confirmed with a sample of 10 occupational therapists representative of the wider sample studied (75% graduated > 5 years) and the AFT demonstrated responsiveness before and after a two day EBP workshop [20]. The 'Modified Fresno test' (MFT) for physical therapists considered all 12 items of the Fresno test, with item stems almost identical to the original test [21]. Two further items were added to the modified test regarding the other pillars of EBP; patient preferences and clinical expertise, with the clinical expertise item removed following poor psychometric performance. Three cohorts of respondents were recruited for this study, first year physical therapy students (novices n = 31), third year Doctor of Physical Therapy students (EBP trained students n = 50) and physical therapy faculty (EBP experts n = 27). The 'Modified Fresno test' for physical therapists demonstrated inter- and intra-rater reliability (inter-rater ICC 0.91, intra-rater ICC 0.95, 0.96), internal consistency (Cronbach's α 0.78) content and discriminative validity (significant difference in total score corresponding to training level *p *< 0.0001) [21].

While the Fresno test has been recognised as the current best available instrument to comprehensively assess cognitive skills of EBP [8], the instrument is not without limitations as noted by developers of two modified versions [20,21]. The Fresno test includes open ended questions requiring short essay style answers. The answers are then assessed using a standard scoring template with points awarded for key components of each answer. The authors of the Fresno test estimate that respondents require up to 60 minutes for completion of the test [9]. The test is therefore lengthy to complete and difficult to score, with training necessary to score the test. The original test was not relevant to health professions outside of medicine, with medical based clinical scenarios, and reference to issues of diagnosis which would be unlikely to be raised in other health professions [11]. The original Fresno test has been modified for single profession use in occupational therapy [20] and physical therapy [21]. Both of the modified Fresno tests have retained the open ended style of questions and complex scoring template of the original test. Given the absence of an instrument specifically developed for students across entry-level health professional programs, there was a need for the development of an instrument based on the Fresno test as a means to assess cognitive EBP skills across a range of entry-level health professional disciplines.

Due to the focus of this study on the assessment of entry-level education (novice rather than postgraduate learners or clinicians), the instrument was intended to evaluate the first three fundamental steps of the EBP process model (ASK, ACQUIRE and APPRAISE), rather than assessment of the fourth step involving the application of knowledge and skills in clinical practice. The aim of this study was to develop an instrument to evaluate entry-level respondents' cognitive skills regarding the research evidence component of EBP that:

• could be used across the health professions;

• was quick to complete and easy to score; and

• was underpinned by a transparent and defensible psychometric testing process.

## Method

The development of the instrument was completed in two stages. The first stage involved the development of the instrument and the second stage comprised the processes used to psychometrically evaluate the instrument.

Ethical approval for the study was obtained from the University of South Australia Human Research Ethics Committee (protocol number P067/08).

### Stage 1: Development of the instrument

Firstly, the research team considered the content of the Fresno test in the drafting of the instrument. The first seven items of the Fresno test were relevant for application to health professions external to medicine and were considered in the development of the K-REC instrument. The remaining Fresno items (8-12) involved advanced statistical calculations (eg predictive values, number needed to treat, risk reduction), and questions about diagnosis and prognosis. While these skills are pertinent to all health professions, the instrument was intended for use with entry-level health professional students with varying degrees of exposure to EBP education (novice learners rather than graduates of professions). Therefore, a choice to focus on what might reasonably be expected to be taught generically across a range of degrees/stages of programs and across disciplines was required. In both forms of the modified Fresno test [20,21], the specific statistical items of the original Fresno test have been altered or removed. The choice was made to limit statistical knowledge items in the instrument to interpretation of metrics of significance and focus upon questions of intervention. Knowledge of levels of evidence was not assessed in the original Fresno test but was considered an essential component and was therefore included.

One clinical scenario template was designed for the K-REC, with specific inclusion of each of the PICO components. The clinical scenario needed to be relevant to a variety of health professional disciplines and was therefore deliberately designed around the topic of a chronic condition (cystic fibrosis) and two possible types of intervention for this condition. The interventions included in the scenario (exercise and breathing exercises) were intentionally chosen to be understood by a number of health professional disciplines (e.g. physiotherapy, occupational therapy, podiatry, human movement).

Given the time and scoring related limitations of the Fresno test, the K-REC was designed to be relatively quick for respondents to complete and easy to score. The K-REC therefore consisted of a combination of short answer, multiple choice and true/false type questions rather than conforming to the open ended short essay answer style of the Fresno test. The final draft of the K-REC instrument was distributed to three senior academics with expertise in research methods and EBP and two entry-level physiotherapy students at the University of South Australia to review the clarity and formatting of the items and instructions. The instrument underwent three rounds of review and minor revision to the wording, instructions and layout of the instrument.

#### The final K-REC instrument

The K-REC instrument was designed to evaluate cognitive skills of EBP, with a combination of items evaluating either knowledge, or skills in the application of knowledge (for example, the application of knowledge to write a relevant research question based on the clinical scenario). The K-REC instrument was intended to assess **entry-level **respondents' cognitive skills regarding the research evidence component of EBP. The instrument therefore covered the first three steps of the five step EBP process model relevant to cognitive skill (ASK, ACQUIRE and APPRAISE), but did not consider the last two steps involving the application of evidence into practice (APPLY and ASSESS) [7]. The final draft of the K-REC consisted of a clinical scenario template and nine items relating to that scenario (Additional file 1).

The 'correct' answers for each item of the K-REC were determined through review of the Fresno test, and consultation and consensus of the research team and senior academics during the development procedure. The two open-ended items (1 and 7) were scored according to the presence or absence of set criteria. For item one, respondents were allocated half a mark for each of the PICO (Population, Intervention, Control or Comparator, Outcome) components provided in their question. In item seven, respondents were required to list four characteristics of randomised controlled trials that would increase their confidence that the research was methodologically sound. Half a mark was awarded for any characteristic that was consistent with the Physiotherapy Evidence Database (PEDro) scale [22] which is an appraisal of methodological bias tool specifically for randomised controlled trials. Two other characteristics of randomised controlled trials were added as correct answers for this item as a result of answers provided by students during the survey development procedure (refer to the K-REC marking guidelines in Additional file 2).

### Stage 2: Pilot study: psychometric evaluation of the K-REC instrument

The aims of Stage 2 of the instrument development process were to explore the practicalities of the survey process (recruitment, dissemination, workload required of participants, and response rate), establish test-retest and inter-rater reliability, investigate the validity of the K-REC, and seek informal feedback from the participants about the appearance, layout and general user friendliness of the instrument.

#### Participants

The recruitment strategy aimed to enlist two main types of participants:

• '***Exposed'***: participants who were representative of the target population for the K-REC and who could be reasonably expected to have a good understanding of EBP through exposure to formal EBP training, and,

• '***Non-exposed'***: participants who could be reasonably expected to have minimal understanding of EBP through minimal or no exposure to formal EBP training.

Third year physiotherapy students (n = 24) were invited to participate as the 'exposed' group. At the time of the study (2008), these students had completed either a mandatory stand-alone EBP course (13 weeks) or an Honours research preparation course (2 weeks and ongoing supervision within a research team) where they were taught and assessed on the different components of EBP. Therefore, these students had prior exposure to formal training and were expected to have a foundation knowledge and understanding of EBP.

Human movement students (n = 89) were invited to participate as the 'non-exposed group'. Rather than compare first and third year students from the same professional discipline (physiotherapy), human movement students were selected as they would have been exposed to similar information technology literacy (databases, searching) and were at the same time point in their entry-level programs as the physiotherapy students (third year). The human movement students were expected to have minimal knowledge of EBP, as at the time of the study these students were not required to enroll or be assessed in any EBP or research training course and were therefore not exposed to any formal standardised EBP or research instruction.

#### Pilot study procedure

The entire group of 24 physiotherapy students was invited to complete the K-REC instrument at a face to face class. Students were requested to write their student identification number on the survey to be used to match the second round of surveys (test-retest). A second administration to the participating physiotherapy students (n = 24) was completed three days after the first testing occasion. The completed surveys from the first testing occasion (n = 24) were independently scored by two raters to establish the inter-rater reliability of the instrument. In order to establish the discriminative validity of the K-REC, the 'non-exposed' cohort of human movement (n = 89) students were invited to participate at a face to face class. The mean item and total K-REC scores of each of the student groups ('exposed' and 'non-exposed') were compared to establish discriminant validity.

#### Data analysis

Test-retest and inter-rater reliability was assessed by using the intraclass correlation coefficient (ICC) for items with interval data or those with ordinal data where the intervals between measurements were assumed as equivalent (item 7 and total scores). The reliability of the remaining items was assessed using Cohen's Kappa coefficient (un-weighted) for categorical data (items 2, 4, 5, 6, 8 and 9) and percentage agreement (items 1 and 3). Agreement of less than 0.50 was classified as poor, between 0.50 and 0.75 as moderate, and greater than 0.75 was classified as a good level of reliability [23].

Discriminative validity was assessed by comparison of the individual item and total scores from the two student groups (human movement and physiotherapy) with calculation of unpaired t tests, and the z-test for comparing proportions (two-tailed) for the differences between the percentage of students achieving a pass (≥ 50% score) mark for each of the survey items in both student groups [24]. Probability values of less than 0.05 were deemed statistically significant.

## Results

The K-REC achieved an overall response rate of 88 per cent (physiotherapy students: 100%, human movement students: 85%). The average completion time for the K-REC was approximately 10 minutes.

### Reliability

All K-REC items and the total score achieved a moderate level of test-retest reliability or above (Table 2). All participating 'exposed' participants (n = 24) completed the retest. A good level of agreement between raters was achieved for the individual items and the total score (Table 2).

**Table 2:** Test-retest and inter-rater reliability of the K-REC instrument

Item no. and content assessed	Test-retest reliability	Inter-rater reliability
1 Research question (PICO)	100%	100%

2 Sources of information	K 0.91 (0.85 - 0.97)	K 1.00

3 Study design knowledge	100%	96%

4 Search strategy (MeSH)	K 0.93 (0.79 - 1.00)	K 1.00

5 Search strategy (Boolean)	K 0.77 (0.54 - 1.00)	K 0.83 (0.44 - 1.22)

6 Critical appraisal	K 0.71 (0.32 - 1.00)	K 1.00

7 Critical appraisal	ICC 0.80	ICC 0.87

8a Research evidence statistics	K 0.86 (0.68 - 1.00)	K 1.00

8b Research evidence statistics	K 0.94 (0.82 - 1.00)	K 1.00

9 Levels of evidence	K 0.62 (0.32 - 0.92)	K 1.00

K-REC total scores	ICC 0.88	ICC 0.97

ICC Intraclass correlation coefficient, K Cohen's Kappa coefficient (95% confidence interval), MeSH Medical subject headings, PICO (Participants, Intervention, Control or Comparator, Outcome)

### Validity

Discriminative validity of the K-REC was determined by the comparison of two student groups, one group with prior exposure to EBP training (expected to have an understanding of EBP process), and one group with no prior exposure to formal EBP training (expected to have minimal understanding of EBP process). The results of the total and individual survey item scores of the K-REC for both groups are shown in Table 3. The mean total score was 4.4 points higher for the 'exposed' students compared to the 'non-exposed' students (8.4 versus 4.2 respectively. Unpaired t tests were performed to investigate the differences between these groups. Seven out of 10 K-REC items (items 8a and 8b were considered separately) showed a significant difference (questions 1, 3, 6, 7, 8a, 8b and 9). The three items (questions 2, 4 and 5) that did not show a significant difference were all related to search strategy. There was a highly significant difference (*p *< 0.0001) in the mean total K-REC scores between the 'exposed' and 'non-exposed' students.

**Table 3:** Comparison of scores obtained from the 'exposed' and 'non-exposed' groups and the percentage of students who achieved a ≥50% pass mark for each K-REC item

Item no. and content	Max. possible score	Student scores mean (SD)		*p *value (unpaired t test)	Percentage of students passing (≥50%)		*p *value (z score, 2 tailed)
		**'Exposed'** **n = 24**	**'Non-exposed'** **n = 76**		**'Exposed'** **n = 24**	**'Non-exposed' n = 76**	

1 Research question (PICO)	2	1.5 (0.0)	1.1 (0.6)	**0.0001**	100	78	**0.005**

2 Sources of information	2	1.3 (0.3)	1.2 (0.5)	0.2729	100	79	**0.006**

3 Study design	1	1.0 (0.0)	0.6 (0.5)	**< 0.0001**	100	55	**< 0.0001**

4 Search strategy (MeSH)	0.5	0.3 (0.3)	0.3 (0.3)	1	50	50	1.00

5 Search strategy (Boolean)	0.5	0.3 (0.3)	0.2 (0.2)	0.1693	54	38	0.08

6 Critical appraisal	1	0.8 (0.4)	0.1 (0.4)	**< 0.0001**	79	14	**< 0.0001**

7 Critical appraisal	2	1.8 (0.4)	0.4 (0.7)	**< 0.0001**	100	24	**< 0.0001**

8a Research evidence statistics	1	0.4 (0.5)	0.2 (0.4)	**0.0453**	42	21	0.018*

8b Research evidence statistics	1	0.4 (0.5)	0.1 (0.3)	**0.0003**	38	8	**< 0.0001**

9 Levels of evidence	1	0.7 (0.3)	0.2 (0.3)	**< 0.0001**	79	26	**< 0.0001**

Mean total score	12	8.4 (1.4) (range 6.5 - 11.0)	4.2 (2.0) (range 1.0 - 11.5)	**< 0.0001**	100	21	**0.005**

MeSH Medical subject headings, PICO (Participants, Intervention, Control or Comparator, Outcome)

*p *values shown in bold type represent a statistically significant difference (p < 0.05)

* not significant after Bonferroni correction

Considering the mean scores of each group of students for each K-REC item, for nine out of 10 (item eight has two sections which were considered separately) instrument items a higher proportion of 'exposed' than 'non-exposed' students passed. A z-test for comparing proportions (two-tailed) was performed to investigate the differences between the groups resulting in a statistically significant difference between groups for seven of the 10 instrument items (Table 3).

The values presented in Table 3 of the percentage of pilot study participants who achieved a pass mark (≥50% score) for each K-REC item and the total scores were a representation of the relative difficulty of each of the items. The relative difficulty of each item ranged from 'difficult' with only 26 per cent of the total pilot study participants passing the first section of the research evidence statistics question (8a) and 15 per cent passing the second section (question 8b), to 'moderate' with 84 per cent of participants achieving a pass mark for question two on the identification of sources of information.

## Discussion

The K-REC met the basic psychometric requirements for reliability, validity and usability of a standard instrument for collecting data on respondents' cognitive skills of EBP. The information gained through testing the instrument on two health professional discipline areas at one institution suggests that if the instrument is disseminated in a similar fashion, a reasonable response rate should be achieved.

There are a variety of instruments available to assess EBP cognitive skills, some of which have been demonstrated to discriminate between novice and expert learners. The issue with pre-existing tools is that for novice learners, the relevance of the scenario questions and a floor effect is likely to be the issue especially with entry-level students in the early years of their programs. The K-REC instrument was designed to assess the cognitive EBP skills of novice learners (entry-level students) irrespective of their professional discipline. That is, the anticipated target audience of this instrument was predominantly entry-level health professional students, who could range from pre-novice (no exposure to EBP principles at all), novice and intermediate EBP learners. While the K-REC could be used for expert learners, we suspect that a ceiling effect is likely to occur which may make it a useful tool for comparison between participants of varying EBP exposure but it is likely to be insensitive to change in EBP experts (which is likely to be the case for most, if not all other EBP knowledge instruments).

The K-REC differed from existing instruments in terms of design, length, content and psychometric properties. The K-REC contains a clinical scenario template that may be relevant to a variety of health professional disciplines and individual items relating to the scenario. The K-REC therefore has the potential to be applicable to a variety of health disciplines, rather than being specific to one discipline. In contrast to the original Fresno test [9], and two modified versions [20,21], the K-REC was designed to be short, easy to complete and disseminate. The short answer and multiple choice design ensured the usability of the instrument as reflected in the short completion times.

The K-REC demonstrated test-retest and inter-rater reliability, content and discriminative validity (Table 4). A follow on study [25] established the responsive validity of the instrument, with the K-REC demonstrating the ability to detect impact of EBP training in a cohort of 77 physiotherapy students (*p *< 0.001, effect size 1.13). When comparing the psychometric properties of the K-REC to the other two instruments developed for allied health (Table 1), the K-REC was the only instrument that established test-retest reliability. The multiple choice and short answer design of the instrument, and the set marking guidelines are likely to have resulted in the high level of inter-rater reliability of the instrument (range: 0.83 to perfect agreement). The K-REC was able to effectively discriminate between those who had prior formal exposure to EBP training and those who had minimal exposure. Due to the lack of a comparator instrument which assessed cognitive EBP skills in the allied health disciplines at the time of the study, convergent validity was unable to be assessed. The subsequent publication of the two modified Fresno tests developed for the allied health disciplines [20,21] provides the ideal opportunity to compare the K-REC to these alternate instruments.

**Table 4:** Summary of the psychometric properties of the K-REC*

Test property	Measure used	Acceptable results	K-REC performance
**Test-retest reliability** (comparison of scores on an initial test to scores by the same participant on a retest) **Inter-rater reliability** (the degree of agreement between two independent raters)	Cohen's Kappa, ICC and percentage agreement	At least a moderate level of agreement (0.50) between testing occasions (test-retest) or raters (inter-rater)	**Test-retest reliability** Each of the items and total scores achieved at least a moderate level of agreement (range: 0.62 to perfect agreement) **Inter-rater reliability** Each of the items and total scores achieved a very good level of agreement between raters (range: 0.83 to perfect agreement)

**Content validity **(instrument covers entire topic of interest)	Expert opinion	Test covers all of the main aspects of EBP	Content and revisions based on experts' suggestions

**Item difficulty **(relative difficulty of each item)	The % of candidates who answer achieve a passing score	A wide range of difficulties allows a test to be used with both 'exposed' and 'not exposed' groups	Ranged from moderate (84% question 2) to difficult (15% question 8b)

**Construct validity **(evidence that the instrument measures the construct that it intends to)	***Discriminative validity*** Mean scores of 'exposed' and 'not-exposed' compared by *t *test	Significant difference, higher 'exposed' student scores	'Not exposed' mean (human movement) was 4.2 and 'exposed' mean (physiotherapy) was 8.4 **(p < 0.0001)**

	% passing for 'exposed' and 'not-exposed' groups compared by the z-test for comparing proportions	Higher % of 'exposed' students passing	For 9 out of 10 instrument items a higher proportion of 'exposed' (physiotherapy students) than 'not exposed' (human movement students) passed. There was a significant difference between groups for 7 of the 10 instrument items.

EBP Evidence based practice

* Responsive validity established in a follow on study [25], with the K-REC demonstrating the ability to detect impact of EBP training in a cohort of 77 physiotherapy students (*p *< 0.001, effect size 1.13).

Developers of instruments assessing EBP knowledge face a number of philosophical and practical issues. While the Sicily Statement clearly conveys the five fundamental steps of EBP, no similar consensus statements exist to guide which specific knowledge and skills might be considered essential to all learners regardless of stage of training (novice or expert) or professional discipline versus optional, advanced or profession specific knowledge. This might explain why there is such a profusion of derivative instruments from the original Fresno test which modify, exclude or add items concerning statistical concepts, scenarios relevant to various professional groups, or focus upon questions of intervention rather than giving equal weight to questions of prognosis or diagnosis. Where instruments have been developed to assess the learning outcomes of specific EBP training courses, it is likely that the key items included within the assessment are reflected within the specific EBP education program. For example, the content of the Fresno test [9] was designed "to assess the effectiveness of a comprehensive evidence based medicine curriculum in the University of California, San Francisco's Fresno family practice residency programme" (pg 319) and as such, the content of the instrument reflected the learning objectives of a specific evidence based medicine course reviewed and revised by family practice residents, faculty and self identified evidence based medicine experts. In contrast, the Adapted Fresno Test [20] was developed to assess change in skills and knowledge after completion of a two day EBP workshop for occupational therapists. The curriculum of the workshop did not include number needed to treat, risk reduction or how to interpret studies of diagnostic accuracy, which were considered to be more suitable for advanced workshops.

While it is clear that entry-level students should be able to critically analyse and evaluate the usefulness of research in disciplines such as physiotherapy and occupational therapy [26,27], there is currently no consensus about what statistical content should be included in entry-level health professional curricula. In medicine, it has been clearly documented that the processes used to teach, facilitate learning and assessment of medical statistics should be student centred and clinically integrated [28,29]. However, there are currently no clear guidelines about exactly what content should be taught in undergraduate medical programs, with studies on statistics training showing differing content between institutions [30,31]. Our intent in developing the K-REC was to include items which could be reasonably expected to be included within entry-level curricula relating to EBP education regardless of year level across a range of health professional programs, the majority of which are targeted at providing health care interventions. Consequently rather than include more advanced statistics (risk ratio, number needed to treat) we opted to include metrics of significance (*p *value, effect size and confidence intervals) and questions relating to intervention (rather than prognosis/diagnosis).

An additional issue concerning development of EBP evaluation instruments is whether the instrument is intended for repeated use with the same group of participants (eg pre and post EBP training). Where the identical instrument is used on repeated occasions with the same group, concerns arise over respondent familiarity with the items and expected answers. In a number of instruments, provision has been made for alternate items to be used on repeated occasions (Berlin: Set A or B, APT: two clinical scenarios, MFT: three clinical scenarios). The clinical scenario for a question of intervention developed for the K-REC was intended to comprise a template where the specific condition, intervention, comparator and outcome could be modified without altering the intent of the questions relating to the scenario (Q1: write a PICO format search question, Q2: identify sources of information likely to provide reliable/valid information Q3: most appropriate design to answer search question etc). For example, the PICO components relevant in the scenario included within this tested version of the K-REC were:

"Jane is a **16 year old girl who has cystic fibrosis **(POPULATION) **and she has recently been admitted to hospital with a chest infection**. Jane normally self treats at home with **breathing exercises **(INTERVENTION) taught to her by a physiotherapist. One of her friends also has **cystic fibrosis **(POPULATION) but she treats herself with **exercise **(COMPARATOR), not breathing exercises. Jane wants to know whether her **lung condition **(OUTCOME) would be more effectively managed with an exercise program. You have no experience of either breathing exercises or exercise programs for cystic fibrosis and are not sure what to recommend."

This scenario could be modified to be relevant to any health professional discipline by altering the PICO components, for example, based on one of the clinical scenarios within the AFT [20], the scenario could be modified to:

"Jane is a **16 year old girl who has a traumatic brain injury (POPULATION)**, currently completing her final year exams and referred to you for memory rehabilitation. Jane has a number of **usual strategies **(INTERVENTION) but one of her friends also has had a **traumatic brain injury **(POPULATION) but she manages her memory issues with a **diary **(COMPARATOR). Jane wants to know whether she would **function better at school **(OUTCOME) with a diary. You have no experience of the usual strategies or diaries for memory problems in traumatic brain injury and are not sure what to recommend."

When the K-REC instrument with the above alternate clinical scenario was completed by a sample of entry-level physiotherapy students (n = 15), the inter-rater reliability of the marking guidelines remained high (2 raters, item 1: 93% agreement, items 2-6 and 8-9: 100%, item 7: ICC 0.97, and total scores: ICC 0.97). The K-REC currently consists of a clinical scenario template, with two 'tested' scenarios. If the instrument were to be applied longitudinally, it would be necessary to develop and test a 'bank' of alternate clinical scenarios. While the option is there for users of the K-REC instrument to modify the clinical scenario template to suit various health professional disciplines, it is important to note that any modified scenario would need to be tested appropriately. It is also worth considering that the clinical scenarios contained in the original Fresno test, two modified versions and the instrument developed in the current study were all based on intervention style questions. The Centre of Evidence Based Medicine Levels of Evidence [32] currently recognises six different types of EBP questions (pre-test probabilities, diagnostic accuracy, prognosis, treatment benefits, treatment harms, and screening). While the original and the modified Fresno test for physical therapy [9,21] included two final open ended items unrelated to the scenarios relating to the identification of the best study design for questions of diagnosis and prognosis, it is interesting to note that none of the instruments contained clinical scenarios or the majority of items relating to different types of questions.

## Limitations

It is possible that the three days between test occasions resulted in a degree of respondent familiarity with the items in the K-REC instrument for the test-retest reliability testing. The choice of how long to wait before retesting knowledge items requires a trade-off between the likelihood of participant recall and learning. In test-retest situations where the intent is to determine stability of respondent's answers, a longer duration is likely to reduce the potential for participants to simply recall the answers they provided during the first test occasion but increases the possibility of ongoing learning of the specific knowledge concepts included in the original test. In the current study, participants were university students currently engaged in courses where it was likely that there would be ongoing exposure to EBP principles and knowledge. The choice of a shorter period between test occasions was selected as a compromise between recall and learning.

The predominant multiple choice question design of the K-REC has both advantages and disadvantages. By providing a list of possible answers, it may encourage respondents to complete the question (rather than omit the question) due to the fact that the 'correct' answer is somewhere in the list of options. The answers in the list may also act to prompt respondents if they are unsure or having difficulty remembering the answer to a particular question. It is possible that if the same items were designed as 'fill in the blank' type questions, respondents may be less likely to guess the answer. It is not possible to know from the data collected in this study whether respondents were influenced by the provision of answers in the multiple choice items in the K-REC.

The items in the K-REC that related to the second step of the EBP process (ACQUIRE) did not discriminate between the groups with differing exposures to formal EBP training (items 2, 4 and 5). The ability to effectively search the literature is an essential step in the EBP process [33]. Identifying sources of information such as databases and journals, and completing an efficient and effective search strategy requires skills and knowledge that are applicable to most areas of health professional training, rather than being specific to EBP. It is likely that the students involved in this study (third year students), had previously obtained skills and knowledge in searching the literature effectively in earlier years of their entry-level programs (eg for assignments), regardless of the presence or absence of formal EBP training. It is also possible that the true/false design of items 4 and 5 may have resulted in an inability of these items to discriminate between leaners who 'know about the concept' of search strategy, as opposed to learners who 'know how to apply' the concept [34]. As the K-REC instrument was designed to evaluate skills and knowledge of EBP, rather than behaviours (actual performance of EBP in practice), the underlying reason for the lack of discrimination found in these items is unclear.

## Implications

The K-REC instrument was designed to evaluate the EBP cognitive skills of novice learners (with a focus on entry-level health professionals). It was therefore appropriate that the testing of the instrument compared the two extremes that could be present in entry-level training of health professionals, that is, those with no exposure to formal EBP training versus those with exposure to formal EBP training. While comparisons between novice, intermediate and expert learners would be useful to undertake with the K-REC instrument, it is unknown whether a ceiling effect might exist for the expert group, rendering this instrument inappropriate for this group.

The Sicily Consensus Statement on EBP [7] highlighted a need for effective training in each of the five steps of EBP, and future research into valid and reliable instruments to evaluate this training. While it seems reasonable in theory to advocate the need for EBP evaluation instruments that cover all of the five steps of EBP, this may be a difficult undertaking within any single instrument. For example, instruments which evaluate cognitive EBP skills such as the Fresno test, and the instrument developed in the current study are not placed to evaluate the application of the evidence into practice (step 4: APPLY), or to promote reflection of individual performance of the entire EBP process (step 5: ASSESS). Rather than aiming to develop instruments which assess all of the five steps of EBP, it may be that instruments are designed for specific purposes, such as the evaluation of EBP theoretical courses (instrument to assess cognitive skills), or the evaluation of EBP in clinical practice (instruments to assess performance based skills and application).

Previous EBP instruments have most commonly contained items evaluating the 'ACQUIRE' and 'APPRAISE' steps of EBP [8]. The ten instruments found in the updated systematic search for the current study (Table 1) were also consistent with this finding. The two instruments that were developed for occupational and physical therapy [20,21] have closely followed the original Fresno test and thus evaluated cognitive skills pertaining to the first three steps of EBP. While the Sicily Statement [7] has recommended that instruments cover the five step process model, it is currently not established which core competencies should be included in each of the EBP steps, and whether these competencies are different between professions. For example, while it may be appropriate for a medical doctor involved in clinical research to understand how to calculate number needed to treat, is this a skill that an allied health professional student needs or should be taught during their entry-level training? The question of which competencies should be included for each of the steps in EBP evaluation instruments and whether this differs between professions or levels of training (eg undergraduate or postgraduate) warrants further exploration.

## Conclusion

The K-REC instrument captures cognitive EBP skills relating to the first three steps of the five step EBP process model (ASK, ACQUIRE, APPRAISE). The instrument has demonstrated very good reliability, and the validity findings show promise in the application of the instrument for evaluating change at an undergraduate health professional level.

## Competing interests

The authors declare that they have no competing interests.

## Authors' contributions

LKL, MTW and TSO all instigated and conceived of the study, and participated in the drafting and editing of the manuscript. All authors read and approved the final version of the manuscript. All authors contributed equally to this work

## Authors' information

LKL is a Lecturer in the School of Health Sciences at the University of South Australia. She has recently completed a PhD in the area of EBP and entry-level physiotherapy education. Her research interests are in health professional education, and the teaching of EBP process and best research evidence to undergraduate students.

MTW is an Associate Professor in the School of Health Sciences at the University of South Australia. Her research interests included EBP education, with particular reference to educational processes for best research evidence, and the sensation of breathlessness in people with chronic pulmonary conditions.

TSO is a Professor in the School of Health Sciences at the University of South Australia. His research interests include the links between time use and health outcomes, mathematical modelling of sports performance, anthropometry and historical trends in fitness, fatness, food intake and sleep of children.

## Pre-publication history

The pre-publication history for this paper can be accessed here:

http://www.biomedcentral.com/1472-6920/11/77/prepub

## Supplementary Material

Additional file 1 **K-REC instrument**. Click here for file

Additional file 2 **K-REC marking guidelines**. Click here for file
