# Supplementary material for: Development and psychometric testing of an instrument to evaluate cognitive skills of evidence based practice in student health professionals
Source: BMC Med Educ. 2011 Oct 3;11:77. doi: 10.1186/1472-6920-11-77 (PMC3196731; doi:10.1186/1472-6920-11-77)
Supplement: Additional file 1 — K-REC instrument. [file 1472-6920-11-77-S1.PDF]

The K-REC instrument

Answer questions 1-9 based on the following clinical scenario. If you are not confident in your answer, please do not guess (select the 'don't know' option).

'Jane is a 16 year old girl who has cystic fibrosis and she has recently been admitted to hospital with a chest infection. Jane normally self-treats at home with breathing exercises taught to her by a physiotherapist. One of her friends also has cystic fibrosis but she treats herself with exercise, not breathing exercises. Jane wants to know whether her lung condition would be more effectively managed with an exercise program. You have no experience of either breathing exercises or exercise programs for cystic fibrosis and are not sure what to recommend.'

1. You have decided to search the literature to help you make a decision about what to recommend. Write a question that will help you organise your literature search (text response). 2 marks

2. Identify four sources of information that you would have the most confidence in providing valid information to help you answer your question. 2 marks

- ☐ General internet search
- ☐ Clinical guidelines
- ☐ Cystic Fibrosis Foundation
- ☐ Lecture notes from your university program
- ☐ Electronic databases (eg Medline, Cinahl)
- ☐ People (eg colleague, expert, lecturer)
- ☐ Medical website (eg medicine.com)
- ☐ Systematic reviews (eg the Cochrane library)
- ☐ Text book (eg Pryor & Prasad (2002) Physiotherapy for Respiratory and Cardiac Problems)
- ☐ Professional association (eg Australian Physiotherapy Association)
- ☐ Peer reviewed journals (eg Chest)
- ☐ Don't Know

3. What type of research design would be the most appropriate to answer the question of whether exercise or breathing exercises provide a better health outcome? (please tick only one box) 1 mark

- ☐ Case study
- ☐ Randomised controlled trial
- ☐ Cross sectional study
- ☐ Cohort study
- ☐ Non-randomised controlled trial
- ☐ Survey
- ☐ Don't know

4. If you completed your literature search on Medline using MeSH (medical subject headings) terms, your search would yield <u>fewer articles</u> than if you conducted a basic search using general terms. ½ mark
TRUE/FALSE/DON'T KNOW

5. If you used the Boolean operator 'OR' in your search, it would reduce the number of citations that your search would produce. ½ mark
TRUE/FALSE/DON'T KNOW

6. You locate an experimental study comparing breathing exercises and an exercise program for cystic fibrosis. How will you determine if the research is methodologically rigorous? (please tick only one box) 1 mark

- ☐ if the study is in a peer reviewed journal if guaranteed that the research is methodologically sound
- ☐ use a critical appraisal tool to appraise the risk of bias
- ☐ discuss the research with a colleague / expert in the field to validate the findings
- ☐ all of the above
- ☐ don't know

7. Your literature search produces two randomised controlled trials. List four characteristics of randomised controlled trials that would increase your confidence that the research methodology was scientifically sound? (text response) 2 marks
8. The findings of the two randomised controlled trials are shown below. Both studies show a significant beneficial effect for the treatment versus control group.<br><br>Study 1 compared breathing exercises and an exercise program in 24 subjects. Sputum volume averaged 1.5g ± 2.0 with the exercise program. There was a significant difference between sputum weights (*p*=0.001, 95% confidence interval 0.5-1.2, effect size 1.2)<br><br>Study 2 compared breathing exercises and an exercise program in 12 subjects. Sputum volume averaged 2.2g ± 0.5 with the exercise program. There was a significant difference between sputum weights (*p*=0.04, 95% confidence interval 0.25-1.5, effect size 2)<br><br>a) Which study shows the more beneficial effect? (please circle the answer) 1 mark<br>STUDY 1 / STUDY 2 / DON'T KNOW<br><br>b) How did you decide which study produced the more beneficial effect? (*please tick only one box*) 1 mark<br>☐ *p* value<br>☐ effect size<br>☐ mean sputum volume<br>☐ confidence intervals<br>☐ I didn't know the answer<br>☐ Other (please specify)
9. Rank the following study designs from 1 to 4 according to a hierarchy of evidence (*with 1 being the design that you have the most confidence in for answering your question and 4 the lowest confidence in answering your question)* 1 mark<br>( ) Case control / cohort study<br>( ) Randomised controlled trial<br>( ) Systematic review (meta-analysis)<br>( ) Review paper based upon expert consensus<br>( ) Don't know
**TOTAL / 12 marks**
