# Supplementary material for: Development and psychometric testing of an instrument to evaluate cognitive skills of evidence based practice in student health professionals
Source: BMC Med Educ. 2011 Oct 3;11:77. doi: 10.1186/1472-6920-11-77 (PMC3196731; doi:10.1186/1472-6920-11-77)
Supplement: Additional file 2 — K-REC marking guidelines. [file 1472-6920-11-77-S2.PDF]

K-REC Marking guidelines

Item	Marking guidelines	Score
1	RESEARCH QUESTION<br>• Participants (cystic fibrosis)<br>• Intervention (exercise)<br>• Control or comparator (breathing exercises)<br>• Outcome (one specific outcome measure related to the condition)<br>**TOTAL for question 1**	½ mark each:<br>☐<br>☐<br>☐<br>☐<br>**/ 2**
2	SEARCH STRATEGY<br>• Clinical guidelines<br>• Electronic databases<br>• Systematic reviews<br>• Peer reviewed journals<br>**TOTAL for question 2**	½ mark each:<br>☐<br>☐<br>☐<br>☐<br>**/ 2**
3	RESEARCH DESIGN<br>Randomised controlled trial<br>**TOTAL for question 3**	1 mark:<br>☐<br>**/ 1**
4	SEARCH STRATEGY<br>• True<br>**TOTAL for question 4**	½ mark:<br>☐<br>**/ ½**
5	SEARCH STRATEGY<br>• False<br>**TOTAL for question 5**	½ mark:<br>☐<br>**/ ½**
6	CRITICAL APPRAISAL<br>• Use a critical appraisal tool to appraise the risk of bias in the study<br>**TOTAL for question 6**	1 mark:<br>☐<br>**/ 1**
7	CRITICAL APPRAISAL<br>½ mark allocated for each of the following characteristics:<br>☐ Clear eligibility criteria ☐ ≥85% of subjects were followed up<br>☐ Random allocation ☐ Blinding of assessors<br>☐ Concealed allocation ☐ Blinding of subjects<br>☐ Similar baseline measures ☐ Blinding of therapists<br>☐ Intention to treat analysis ☐ Adequate sample size / power calc<br>☐ Between group statistical comparison ☐ Reliable and valid outcomes<br>☐ Point measures and measures of variability<br>**TOTAL for question 7**	2 marks<br><br><br><br><br><br><br><br><br>**/ 2**
8	RESEARCH EVIDENCE STATISTICS<br>• 8a Study 2<br>• 8b Effect size<br>**TOTAL for question 8**	1 mark each:<br>☐<br>☐<br>**/ 2**
9	LEVELS OF EVIDENCE<br>(1) Systematic reviews<br>(2) Randomised controlled trial<br>(3) Case control / cohort study<br>(4) Review paper based upon expert consensus<br>**TOTAL for question 9**	¼ mark each:<br>☐<br>☐<br>☐<br>☐<br>**/ 1**
**K-REC TOTAL**		**/ 12**
